# Comparative effectiveness and safety of eplerenone and spironolactone in patients with heart failure: a systematic review and meta-analysis

**DOI:** 10.1186/s12872-024-04103-7

**Published:** 2024-09-13

**Authors:** Ahmed Elshahat, Ahmed Mansour, Mohamed Ellabban, Ahmed Diaa, Atef Hassan, Ahmed Fawzy, Omar Abdulrahman Saad, Moaz Abouelmagd, Mahmoud Eid, Ahmed Elaraby, Mohamed Hamouda Elkasaby, Ahmed Abdelaziz

**Affiliations:** 1 Medical Research Group of Egypt (MRGE), Negida Academy, Arlington, MA USA; 2 Faculty of Medicine, Al-Azhar University, Cairo, Egypt; 3 Faculty of Medicine, Cairo University, Cairo, Egypt

**Keywords:** Mineralocorticoid receptor antagonist, MRA, Eplerenone, Spironolactone, Heart failure

## Abstract

**Background:**

Eplerenone and spironolactone, recognized as mineralocorticoid receptor antagonists (MRAs), have been reported to improve clinical prognosis among individuals diagnosed with heart failure (HF). However, the difference in the clinical effects between eplerenone and spironolactone in individuals with HF remains uncertain. We aimed to assess the impact of eplerenone compared to spironolactone on clinical outcomes within the HF population.

**Methods:**

An extensive search was executed in several databases (PubMed, Web of Science, Scopus, Cochrane Library). All relevant studies evaluating eplerenone compared to spironolactone in patients with HF were included. Dichotomous data were pooled as Hazard ratio (HR) or Risk ratio (RR) with a 95% confidence interval (CI). Our main outcome was all-cause mortality. Secondary outcomes included death from cardiovascular causes, treatment withdrawal, and gynecomastia.

**Results:**

Ten studies, comprising 21,930 HF individuals, were included in our investigation. Eplerenone showed a lower risk of all-cause mortality (HR = 0.78, 95%CI [0.64 to 0.94], *P* = 0.009) and cardiovascular mortality (HR = 0.54, 95%CI [0.39, 0.74], *P* = 0.0001) compared to spironolactone. Furthermore, eplerenone exhibited a reduced risk of treatment withdrawal (RR = 0.69, 95% CI [0.62, 0.78], *P* = 0.0001) and gynecomastia (RR = 0.07, 95% CI [0.02 to 0.31], *P* = 0.0001) than spironolactone.

**Conclusion:**

Eplerenone revealed lower all-cause and cardiovascular mortality events in comparison to spironolactone. Moreover, eplerenone was associated with lower gynecomastia and treatment withdrawal events compared to spironolactone. Further well-designed randomized controlled trials are still warranted better to identify the clinical differences between eplerenone and spironolactone.

**Trial registration:**

Protocol registration: https://doi.org/10.17605/OSF.IO/VNMGK

**Supplementary Information:**

The online version contains supplementary material available at 10.1186/s12872-024-04103-7.

## Introduction

Heart failure (HF) is a significant medical issue identified by the heart's impaired ability to pump blood efficiently throughout the entire body. Its prevalence increases with age, affecting around 64 million individuals worldwide. HF is known as one of the most frequent causes of hospitalizations across the world, representing 1–2% of all hospital admissions in the United States and Europe [1]. Therefore, HF has become a major public health problem encouraging us to utilize evidence-based strategies to reduce the risk of HF and limit hospitalizations [2]. The cornerstones of managing heart failure include alleviating symptoms and patients' quality of life, delaying the progression of cardiac and peripheral disorders, and decreasing mortality rates [3].

Based on the most recent recommendations from the American College of Cardiology (ACC), it is recommended to prescribe mineralocorticoid receptor antagonists (MRAs) to all symptomatic heart failure with reduced ejection fraction (HFrEF) patients who do not have contraindications [3]. The ACC guidelines also suggested that an MRA should be considered for individuals with heart failure and mildly reduced ejection fraction (HFmrEF), especially for extremely lower ejection fraction patients. The addition of aldosterone antagonists to the pharmacological treatment of HF has demonstrated significant reductions in hospitalization, overall mortality, and sudden cardiac death instances [4–8].

Nowadays, two of the most prescribed MRAs are eplerenone and spironolactone. Although these two drugs are almost acting on the same biological receptor (mineralocorticoid receptor), their pharmacokinetics are markedly different. Eplerenone is more selective as an MRA than spironolactone, having a higher affinity for the mineralocorticoid receptor and is less likely to interact with other steroid hormone receptors. This selectivity contributes to eplerenone's better tolerability and minimizes the risk of side effects associated with off-target receptor interactions. Additionally, this selectivity is believed to contribute to eplerenone's superiority over spironolactone in improving HF outcomes [9]. The current literature showed conflicting results regarding the clinical effect of eplerenone compared to spironolactone in HF patients, with some studies favoring eplerenone over spironolactone and others not finding any substantial differences between the two drugs [10–12]. To our current knowledge, this study represents the first comprehensive systematic review and meta-analysis that compares eplerenone versus spironolactone in patients with HF, focusing on their impact on efficacy outcomes and safety profiles. This study aims to address the existing conflicting knowledge gap and offer valuable insights to aid clinical decision-making and guide future research in HF management.

## Methods

The Cochrane Handbook rules were followed throughout the execution of our study [13]. Consistent adherence to PRISMA statement guidelines was maintained while reporting this study [14]. The protocol for this study had been registered in the open science framework (OSF) (Registration link: https://doi.org/10.17605/OSF.IO/VNMGK).

### Eligibility requirements

All the relevant studies that directly compared eplerenone and spironolactone in HF patients, with no restriction to specific ejection fraction type or study design, were included in our investigation. Outcomes were classified as efficacy, tolerability, and safety outcomes. The efficacy outcomes were all-cause mortality, cardiovascular mortality, cardiovascular mortality or hospitalization, and HF hospitalization. Tolerability outcomes were the rates of treatment withdrawal and crossover. The safety outcomes were gynecomastia, hyperkalemia, renal failure, and hypotension. Duplicate articles, reviews, editorial letters, and conference abstracts were excluded.

### Determining data sources and search approach

PubMed, Web of Science, SCOPUS, and Cochrane Central were rigorously searched from inception until March 2024. Our search strategy contained a mixture of keywords related to eplerenone, such as “Inspra” and “Selara,” spironolactone, like “Spiractin” and “verospirone,” and heart failure, such as “Cardiac failure.” In-depth information regarding the search methodology for each database is outlined in Supplementary Table 1*.*

### Study selection and data extraction

Using the Rayyan platform, firstly, two independent authors examined the titles and abstracts [15]. Two independent authors assessed full texts using Google Sheets according to the previously established criteria. A third reviewer addressed any conflicts among the review authors. Duplicate papers were excluded using Endnote. (Clarivate Analytics, PA, USA).

Two blinded authors independently retrieved the relevant data from the included papers. Summary data included, e.g., study design, country, inclusion criteria, follow-up duration, and main findings of each included study. Baseline data included e.g., Age, body mass index, gender, comorbidity, and Laboratory results. Any conflict was addressed through a discussion with a third author.

### Quality assessment

Two researchers independently used the Cochrane risk of bias two (ROB 2) tool to evaluate the quality of randomized controlled trials (RCTs) [16]. ROB 2 tool assesses the risk of bias in several domains such as randomization process, deviation from the intended intervention, missing outcome data, outcome measurement, selection of the reported results, and other potential sources of bias. Furthermore, two blinded authors assessed the quality of the included observational studies utilizing the Newcastle–Ottawa-Scale (NOS) [17]. NOS evaluates the quality of the observational papers in three main domains: population selection, comparability between the two cohorts, and outcome assessment. A third author addressed any disagreements between the review authors.

### Statistical analysis

RevMan (v5.3) was used to perform the statistical analysis (5). With a 95% confidence interval (CI), the risk ratio (RR) or hazard ratio (HR) was utilized to pool the dichotomous outcomes. A random-effect meta-analysis model was utilized throughout our primary analysis. A chi-square p-value less than 0.1 or I-square more than 50% was considered a sign of significant heterogeneity.

### Subgroup analysis

We grouped the analysis based on study design as RCT and observational studies and HF type as HFrEF and HFmrEF according to the definitions of recent ACC guidelines [3].

### Evidence robustness

To ensure the pooled effect estimate in our study was not solely determined by any individual study, we conducted a certainty assessment using the leave-one-out test in multiple scenarios, eliminating one study on each examination.

### Publication bias

Due to an insufficient number of studies in any outcome in the meta-analysis, we were unable to evaluate the publication bias [18].

## Results

### Search results

**Fig. 1:**
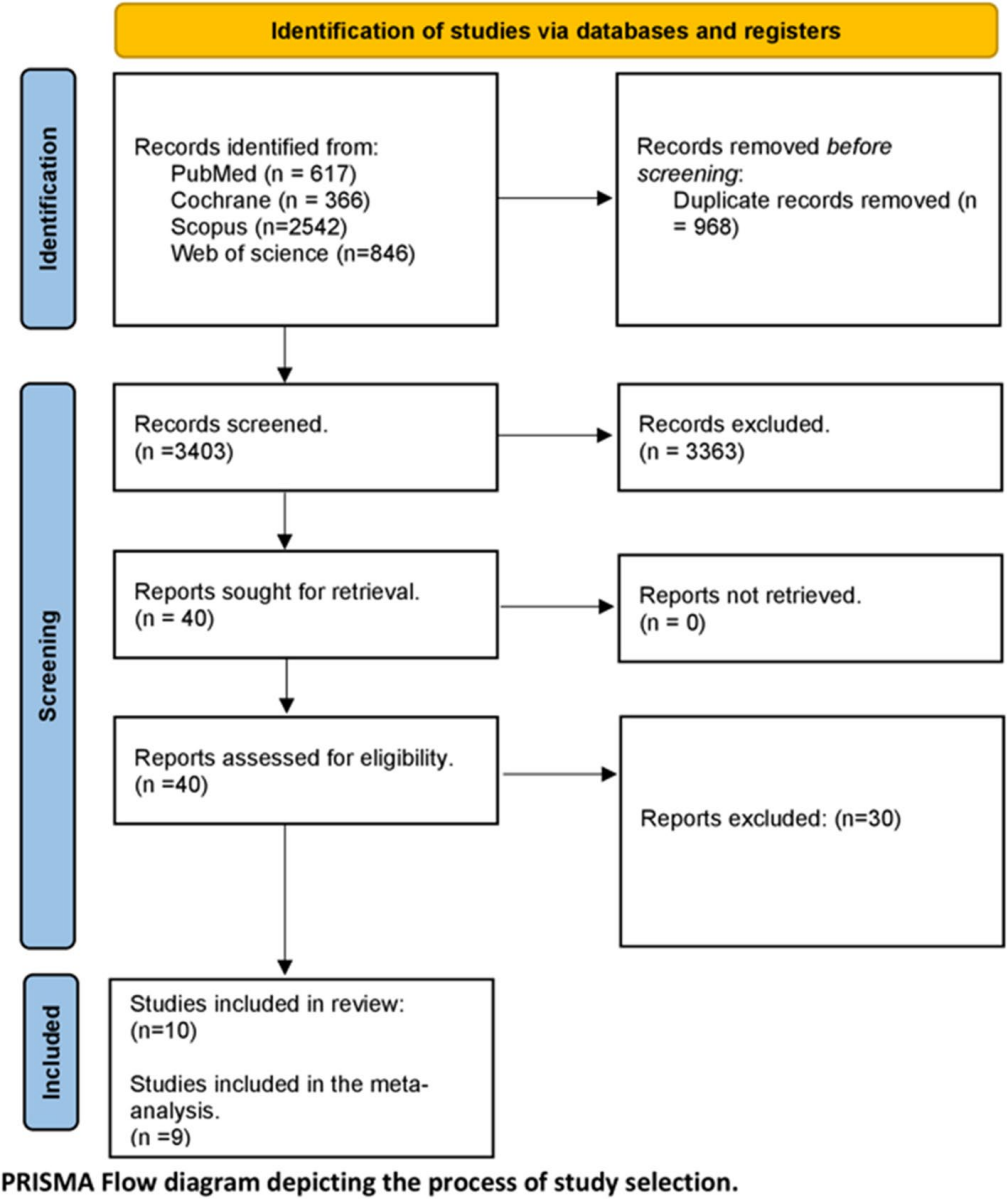
PRISMA flow diagram of the literature search results

We initially identified 4371 records through our literature search, which were reduced to 3403 records after removing duplicates using EndNote (Clarivate Analytics, Philadelphia, United States). After full-text screening and a manual search of reference lists from selected studies, we finally included ten studies. Figure 1 illustrates the PRISMA flow diagram of the study selection process.

### Studies feature

Our study finally encompassed ten studies, with a total of 21,930 HF patients treated with either eplerenone or spironolactone [10–12, 19–25]. Four studies were RCTs, and six were observational studies. These studies were carried out in several countries, such as Canada, Germany, Japan, Denmark, and the USA. The mean age of participants was 58.1, ranging from 53 to 71 years, including 12,749 male participants. The mean follow-up duration was 20 months. The summary and baseline properties of the participants of the studies are presented in Tables 1 and 2 respectively.

**Table 1:** Summary characteristics of the included studies

Study ID	Study design	Country	Total participants	Eplerenone dose (mg)	Spironolactone dose (mg)	Heart failure type	Inclusion criteria	Follow up duration (Months)	Conclusion
Ferreira 2017 [19]	observational	11 international European countries	585	-	-	HFrEF	“Patients aged ≥ 18 years, experiencing symptoms of either new-onset or worsening heart failure with LVEF ≤ 35%, BNP and/or NT-proBNP plasma levels > 400 pg/mL or > 2000 pg/mL, respectively.”	21	“Patients with higher left ventricular ejection fraction and NYHA class III/IV were more prone to MRA discontinuation. Inadequate MRA prescriptions and frequent discontinuations hinder conclusive outcome inferences.”
Korol 2018 [20]	RCT	Canada	62	50	25	HFrEF	“Patients aged ≥ 18 years, diagnosed with heart failure with LVEF ≤ 40%, experiencing symptoms of functional class II-IV according to the New York Heart Association (NYHA), and diagnosed with type II diabetes.”	4	“In patients with HFrEF and coexisting glucose intolerance or diabetes, the study demonstrated that eplerenone did not show a significantly beneficial effect on glucose homeostasis compared to spironolactone.”
Larsson 2023 [21]	observational	Denmark	7493	25 and 50	12.5, 25, 37.5, and 50	HFrEF	“Patients aged 18–85 years, who started treatment with either eplerenone or spironolactone from the time of heart failure diagnosis until 180 days into the study.”	24	“No significant differences in clinical outcomes were noted between the initiation of eplerenone and spironolactone. However, spironolactone-treated patients exhibited more frequent treatment withdrawal and lower daily drug dosages.”
Margolis 2010 [22]	observational	USA	12,550	-	-	HF with no specification	“patients aged ≥ 50, newly prescribed spironolactone or eplerenone, diagnosed with heart failure within 12 months of prescription, and followed up for at least 6 months (12 months for adherence assessment) to evaluate outcomes.”	20	“Eplerenone, compared to spironolactone, exhibited higher compliance and persistence, as well as fewer hospitalizations over the long term in heart failure patients. This makes it an effective alternative for promoting adherence to aldosterone blockade.”
Martínez 2022 [10]	observational	Spain	586	28٫4	26٫9	HFrEF	“Chronic HF patients, with left ventricular ejection fraction < 40% treated with eplerenone and spironolactone.”	48	“Eplerenone, in comparison to spironolactone, demonstrated no statistically significant difference in cardiovascular death or heart failure hospitalization. However, it did reveal a statistically significant reduction in both cardiovascular mortality and all-cause mortality.”
Nabati 2021 [23]	RCT	Iran	77	25	25	HFrEF	“Patients with heart failure, characterized by a left ventricular ejection fraction ≤ 35%, and presenting with NYHA functional class III-IV symptoms.”	6	“The study suggested that incorporating eplerenone into optimal heart failure therapy may lead to greater improvements in echocardiographic measures of left ventricular function compared to spironolactone in symptomatic patients with new-onset systolic heart failure.”
Naser 2023 [11]	RCT	Bosnia and Herzegovi	142	-	-	HFrEF	“Patients aged ≥ 18 years, diagnosed with heart failure with LVEF ≤ 40%, experiencing symptoms of functional class II-III-IV according to the New York Heart Association (NYHA).”	21	“The use of eplerenone in individuals with chronic heart failure has demonstrated positive impacts on cardiac remodeling parameters and significant decreases in cardiovascular and overall mortality when compared to those administered spironolactone.”
Schupp 2022 [24]	observational	Germany	148	27	34	HFmrEF	“HF patients with left ventricular ejection fraction <45% .”	36	“Among patients with ventricular tachyarrhythmias, the administration of MRA (eplerenone and spironolactone) did not demonstrate a significant improvement in overall mortality at the three-year mark.”
Yamaji 2010 [25]	RCT	Jaban	107	50	25	HFmrEF	“Patients with CHF were defined as patients who had previously been admitted to the hospital for management of CHF due to systolic heart failure, which was defined as left ventricular ejection fraction (LVEF) b45% by 2-dimensional echocardiography or ventriculography using contrast medium or radioisotope, or had already been receiving treatment for CHF (mean duration for 5.4 years).”	6	“In chronic heart failure patients, eplerenone, a selective mineralocorticoid receptor blocker, displayed superiority over spironolactone, notably in terms of metabolic effects, especially HbA1c.”
Yamamoto 2019 [12]	observational	Japan	180	25	25 and 50	HFmrEF	“Patients who were hospitalized for acute decompensated HF”	12	“Eplerenone and spironolactone exhibit similar influences on cardiovascular outcomes and safety among heart failure patients.”

*Abbreviations*: *RCT* Randomized controlled trial, *mg* Milligram, *HF* Heart failure, *HFrEF* Heart failure with reduced ejection fraction, *HFmrEF* Heart failure with mildly reduced ejection fraction, *LVEF* Left ventricular ejection fraction, *NYHA* New York Heart Association, *BNP* Brain natriuretic peptide, *NT- proBNP* N-terminal pro–B-type natriuretic peptide

**Table 2:** Baseline characteristics of study population

**Study ID**	**Arms**	**Age, Mean (SD)**	**Male, N (%)**	**Smoking, N (%)**	**BMI, (kg/m2) Mean (SD)**	**Systolic blood pressure, (mmHg) Mean (SD)**	**LVEF, (%) Mean (SD)**	**IHD**	**Comorbidity, N (%)**									**Medical therapy, N (%)**				
									**Dyslipidemia**	**PVD**	**HTN **	**DM**	**AF**	**CKD**	**Stroke **	**COPD**	**Loop diuretics**	**SGLT2-inhibitors**	**Beta blockers**	**Statins**	**Sacubitril-valsartan**	**Digoxin**
Ferreira 2017 [19]	**Eplerenone**	-	-	-	-	-	-	-	-	-	-	-	-	-	-	-	**-**	**-**	**-**	**-**	**-**	**-**
	**Spironolactone**	-	-	-	-	-	-	-	-	-	-	-	-	-	-	-	**-**	**-**	**-**	**-**	**-**	**-**
Korol 2018 [20]	**Eplerenone**	67.4(11)	27(90)	-	30.2(5.3)	114.3(15.3)	32.1667(11.676)	22 (73.3)	-	-	23 (76.6)	21 (70)	15 (50)	-	2 (6.7)	-	25(83.3)	-	29(96.7)	-	-	6(20)
	**Spironolactone**	64(8.2)	30(93.8)	-	30.6(4.3)	112.1(12.4)	30.667(7.76)	23 (71.9)	-	-	27 (84.4)	22 (68.6)	15 (46.9)	-	9 (28.1)	-	26(81.3)	-	31(96.9)	-	-	15(46.9)
Larsson 2023 [21]	**Eplerenone**	64.667(14.12)	591(90.5)	-	-	-	-	296 (45.3)	-	56 (8.6)	-	134 (20.5)	-	21(3.2)	49 (7.5)	47 (7.2)	441(67.5)	36(5.5)	653(100)	418(64)	59(9)	49(7.5)
	**Spironolactone**	68(12.63)	4651(68)	-	-	-	-	2805 (41)	-	652 (9.5)	-	1481 (21.7)	-	290 (4.2)	617 (9)	776 (11.3)	4980(72.8)	253(3.7)	6840(100)	3971(58.1)	348(5.1)	884(12.9)
Margolis 2010 [22]	**Eplerenone**	72(10.12)	415(73)	-	-	-	-	201 (35.39)	-	-	-	202 (36)	179(32)	45(8)	-	-	431(76)	-	447(79)	373(66)	-	203(36)
	**Spironolactone**	74(10.68)	6513(54)	-	-	-	-	3768 (31.45)	-	-	-	4191 (35)	4316 (36)	892(7)	-	-	9131(76)	-	7867(66)	5981(50)	-	3461(29)
Martínez 2022 [10]	**Eplerenone**	61.5(10.2)	234(79.7)	209(71.3)	-	116(20)	27.5(7)	146 (49.8)	164(56)	29 (9.9)	150 (51.2)	98 (33.4)	60 (20.5)	-	21 (7.2)	29 (9.9)	258(88.1)	-	280(95.6)	-	43(14.7)	29(9.9)
	**Spironolactone**	61.2(11.7)	234(79.7)	208(71)	-	116(19)	27٫4	145 (49.5)	146(54.6)	25 (8.5)	145 (49.5)	96 (32.8)	66 (22.5)	-	25 (8.5)	31(10.6)	255(87)	-	281(95.9)	-	42(14.3)	24(8.2)
Nabati 2021 [23]	**Eplerenone**	56.30(15.05)	16(48)	4 (12)	-	-	-	-	9 (27)	-	17 (51)	6 (18)	2 (6)	-	-	-	-	-	-	-	-	-
	**Spironolactone**	53.94(17.60)	12(39)	5 (16)	-	-	-	-	8 (26)	-	12 (39)	9 (29)	5 (16)	-	-	-	-	-	-	-	-	-
Naser 2023 [11]	**Eplerenone**	65.8(7.2)	49(68)	-	26.5(4.3)	114(12)	-	25 (35.1)	44(61.4)	-	46(64.5)	30 (41.4)	18 (26.1)	25 (34.8)	8 (11.3)	12 (17.1)	65(92.1)	19(26.4)	63(88.5)	-	-	21(29.8)
	**Spironolactone**	65.7(7.1)	49(69)	-	26.8(4.2)	115(6)	-	24 (34.5)	43(60.9)	-	46(64.8)	30 (42.2)	19 (27.2)	25 (34.7)	8 (10.9)	11 (15.6)	65(91.9)	19(26.1)	63(89.3)	-	-	20(29.8)
Schupp 2022 [24]	**Eplerenone**	58.3(42)	-	-	-	-	-	-	-	-	-	-	-	-	-	-	-	-	-	-	-	-
	**Spironolactone**	58.3(42)	-	-	-	-	-	-	-	-	-	-	-	-	-	-	-	-	-	-	-	-
Yamaji 2010 [25]	**Eplerenone**	66.3(1.2)	20(28)	-	23.6(1.0)	131.1(2.0)	46.5(0.9)	13(19)	49 (67.1)	-	45 (61.6)	28 (38.4)	-	-	-	-	24 (32.9)	-	43 (58.9)	41 (56.2)	-	-
	**Spironolactone**	63.9(1.9)	27(80)	-	23.6(1.2)	128.4(3.3)	45.7(1.5)	5(14)	22 (64.7)	-	19 (55.9)	17 (50)	-	-	-	-	12 (35.3)	-	23 (67.6)	20 (58.8)	-	-
Yamamoto 2019 [12]	**Eplerenone**	71(11)	61 (68)	53 (59)	22.9(4.7)	148(33)	45(16)	56(62)	-	-	50(56)	43(48)	34(38)	-	-	-	73(81)	-	72(80)	34(38)	-	-
	**Spironolactone**	71(14)	54 (60)	50 (56)	23.6(4.1)	147(39)	46(15)	56(62)	-	-	52(60)	47(52)	35(39)	-	-	-	81(90)	-	74(82)	35(39)	-	-

*Abbreviations*: *N*  Number, *SD* Standard deviation, *BMI* Body mass index, *LVEF* Left ventricular ejection fraction, *IHD* Ischemic heart disease, *PVD* Peripheral vascular disease, *DM* Diabetes mellitus, *AF* Atrial fibrillation, *HTN* Hypertension, *CKD* Chronic kidney disease, *COPD* Chronic obstructive pulmonary disease, *SGLT2* Sodium-glucose-Co-Transporter-2

### Quality assessment

**Fig. 2:**
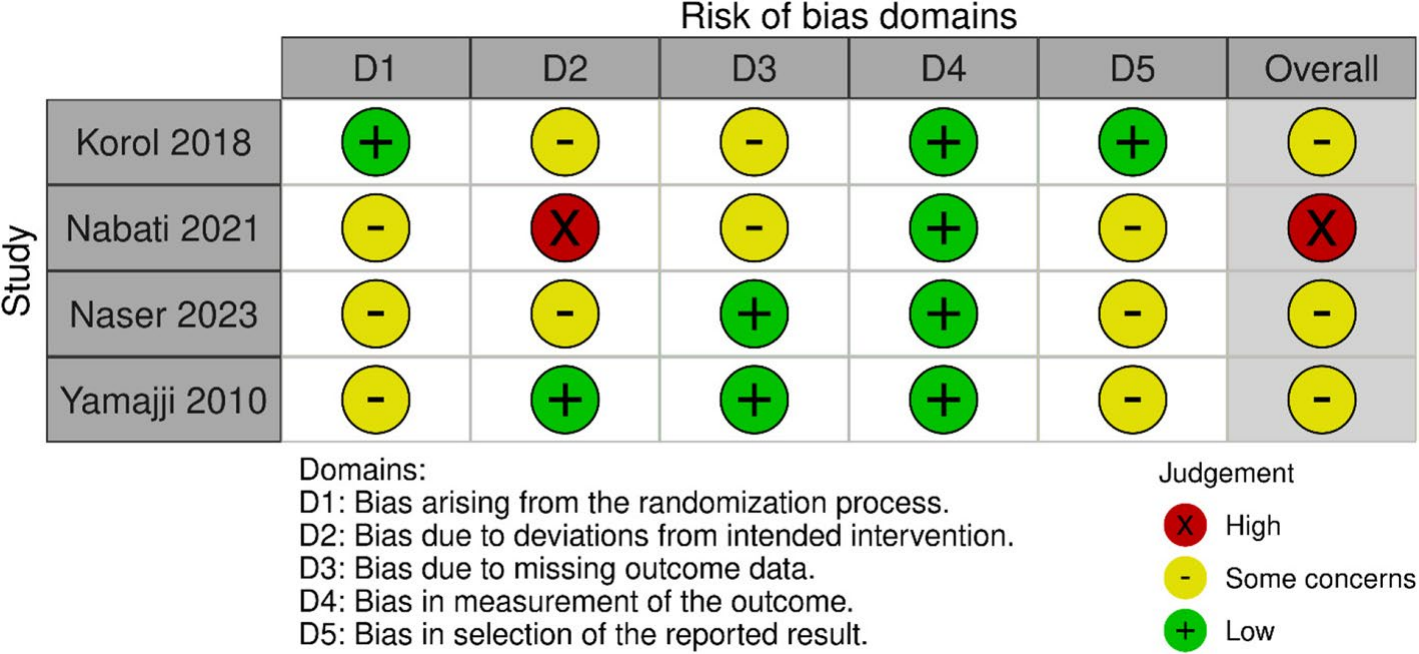
Risk of bias graph

Utilizing the RoB2 tool, Three RCTs exhibited a moderate risk of bias, primarily due to unexplained issues in the randomization process and the selection process of reported results. One RCT was identified as having a high bias risk due to unexplained deviations from intended interventions [23]. The detailed risk of bias for each RCT is illustrated in Fig. 2.

Four observational studies, evaluated using the NOS, showed good quality, while two were classified as fair quality. The lower rating of those two studies was primarily due to issues with comparability in the assessment domain. In-depth information regaridng the risk of bias for observational studies is presented in Table 3.

**Table 3:** Risk of bias assessment for observational studies using New-Castle-Ottawa Scale

Study ID	Representativeness of the exposed cohort	Selection of the non-exposed cohort	Ascertainment of exposure	Demonstration that outcome of interest was not present at start of study	Comparability of cohorts on the basis of the design or analysis	Assessment of outcome	Was follow-up long enough for outcomes to occur	Adequacy of follow-up of cohorts	Quality Score
Yamamoto 2019 [12]	*	*	*		**	*	*	*	Good
Schupp 2022 [24]	*	*	*			*	*	*	Fair
Ferreira 2017 [19]	*	*	*		*	*	*		Good
Larsson 2023 [21]	*	*	*			*	*	*	Fair
Margolis 2010 [22]	*	*	*		*	*	*		Good
*Martínez 2022 *[10]	*	*	*		**	*	*	*	*Good*

## Study outcomes

### Efficacy outcomes

#### All-cause mortality

**Fig. 3:**
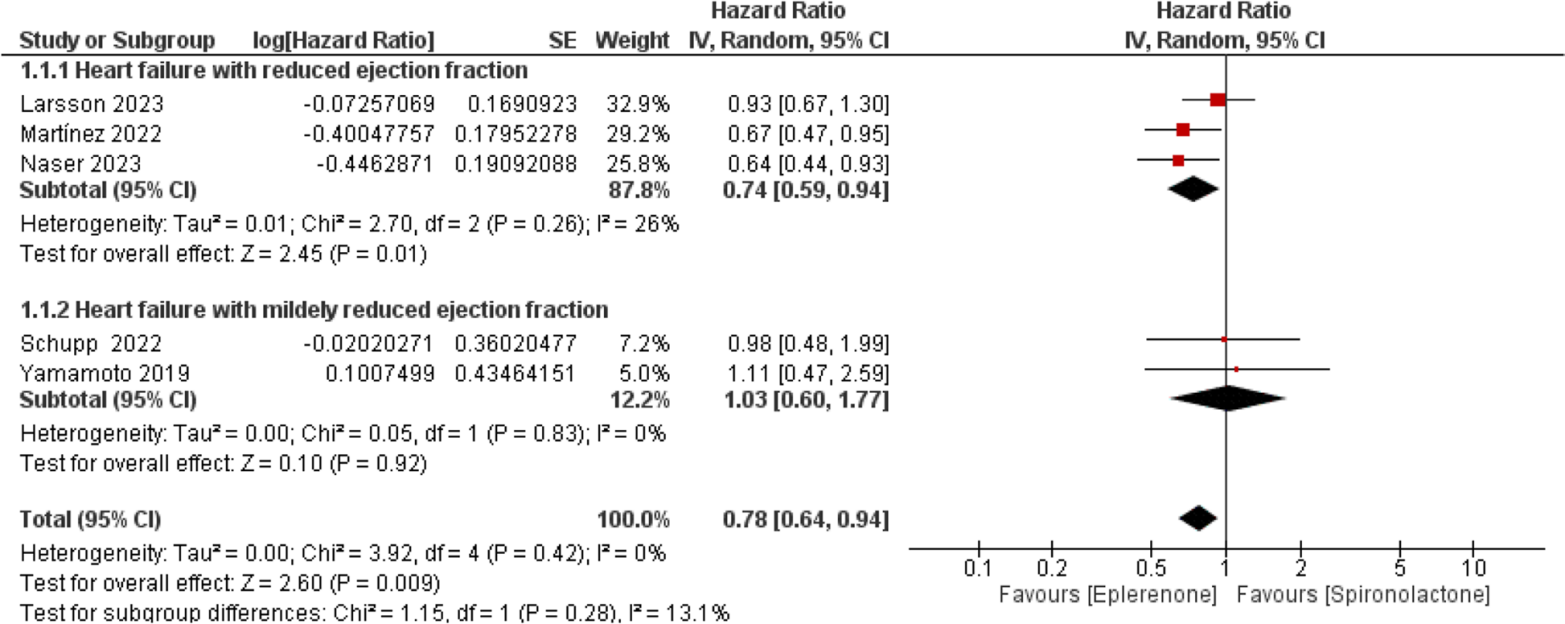
Forest plot of all-cause mortality, SE: Standard error, IV: Inverse variance, CI: Confidence interval

Five studies reported all-cause mortality events. The pooled HR revealed that Eplerenone exhibited lower mortality instances compared to spironolactone (HR = 0.78; 95% CI [0.64, 0.94], *P* = 0.009). The pooled studies were not heterogeneous (I^2^ = 0%, *P* = 0.42), as outlined in Fig. 3.

Eplerenone showed lower mortality rates than spironolactone in patients with HFrEF (HR = 0.74; 95% CI [0.59, 0.94], *P* = 0.01). Also, the HR didn’t favor either of the drugs in patients with HFmrEF (HR = 1.03; 95% CI [0.60, 1.77], *P* = 0.92), as presented in Fig. 3.

Moreover**,** within observational studies, eplerenone showed comparable mortality rates when compared to spironolactone (HR = 0.83; 95% CI [0.67, 1.04], *P* = 0.10). One RCT reported all-cause mortality and showed lower mortality rates with eplerenone than spironolactone (HR = 0.64; 95% CI [0.44, 0.93], *P* = 0.02), as illustrated in Supplementary Fig. 1*.*

The leave-one-out analysis was conducted and showed that after excluding Martinez et al. [10] and Naser et al. studies [11], the results were not significant: (HR = 0.83; 95% CI [0.66, 1.04], P = 0.10) and (HR = 0.83; 95% CI [0.67, 1.04], *P* = 0.10), respectively (see Supplementary Fig. 2 and Supplementary Fig. 3).

### Cardiovascular mortality

**Fig. 4:**
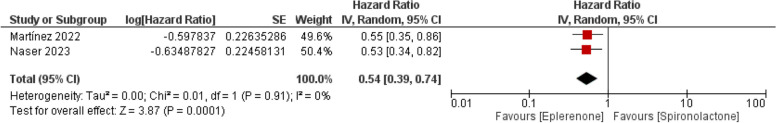
Forest plot of the cardiovascular mortality, SE: Standard error, IV: Inverse-variance, CI: Confidence interval

Only two studies [10, 11] conducted within the HFrEF population accessed cardiovascular mortality rates. The overall HR favored eplerenone over spironolactone in decreasing the cardiovascular mortality rates in individuals with HFrEF (HR = 0.54; 95% CI [0.39, 0.74], *P* = 0.001), and pooled results were homogenous (I^2^ = 0%, *P* = 0.91) as outlined in Fig. 4.

### Cardiovascular mortality or hospitalization

**Fig. 5:**
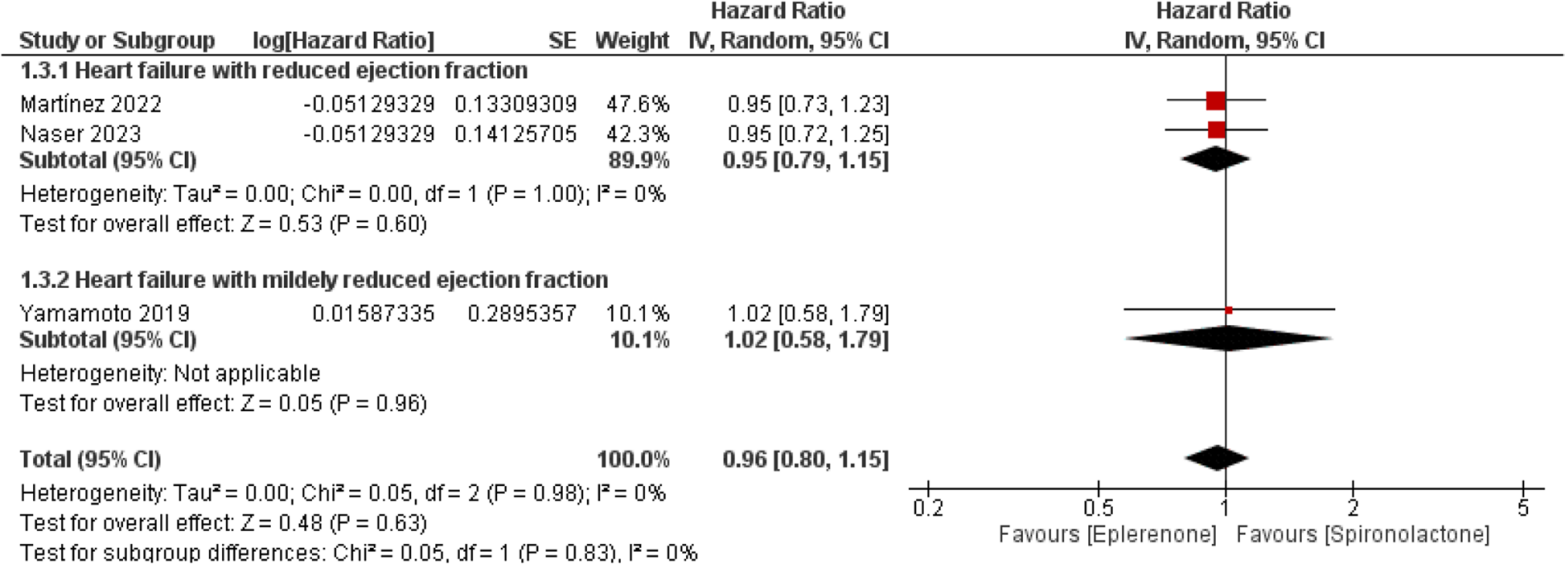
Forest plot of the composite outcome of cardiovascular mortality or hospitalization, SE: Standard error, IV: Inverse-variance, CI: Confidence interval

The pooled HR did not show any preference for either eplerenone or spironolactone (HR = 0.96; 95% CI [0.80, 1.15], *P* = 0.63). Pooled studies showed a non-significant heterogeneity (I^2^ = 0%, *P* = 0.98), as presented in Fig. 5.

Also**,** based on the Heart failure type subgroup, Eplerenone showed no superiority over spironolactone in patients with HFrEF or HFmrEF (HR = 0.95; 95% CI [0.79, 1.15], *P* = 0.60) or (HR = 1.02; 95% CI [0.58, 1.79], *P* = 0.96), respectively, as illustrated in Fig. 5*.*

The pooled effect size remains statistically insignificant within observational and RCT studies (HR = 0.96; 95% CI [0.76, 1.22], *P* = 0.74), (HR = 0.95; 95% CI [0.72, 1.25], *P* = 0.72) respectively as illustrated in Supplementary Fig. 4.

### HF hospitalization

**Fig. 6:**
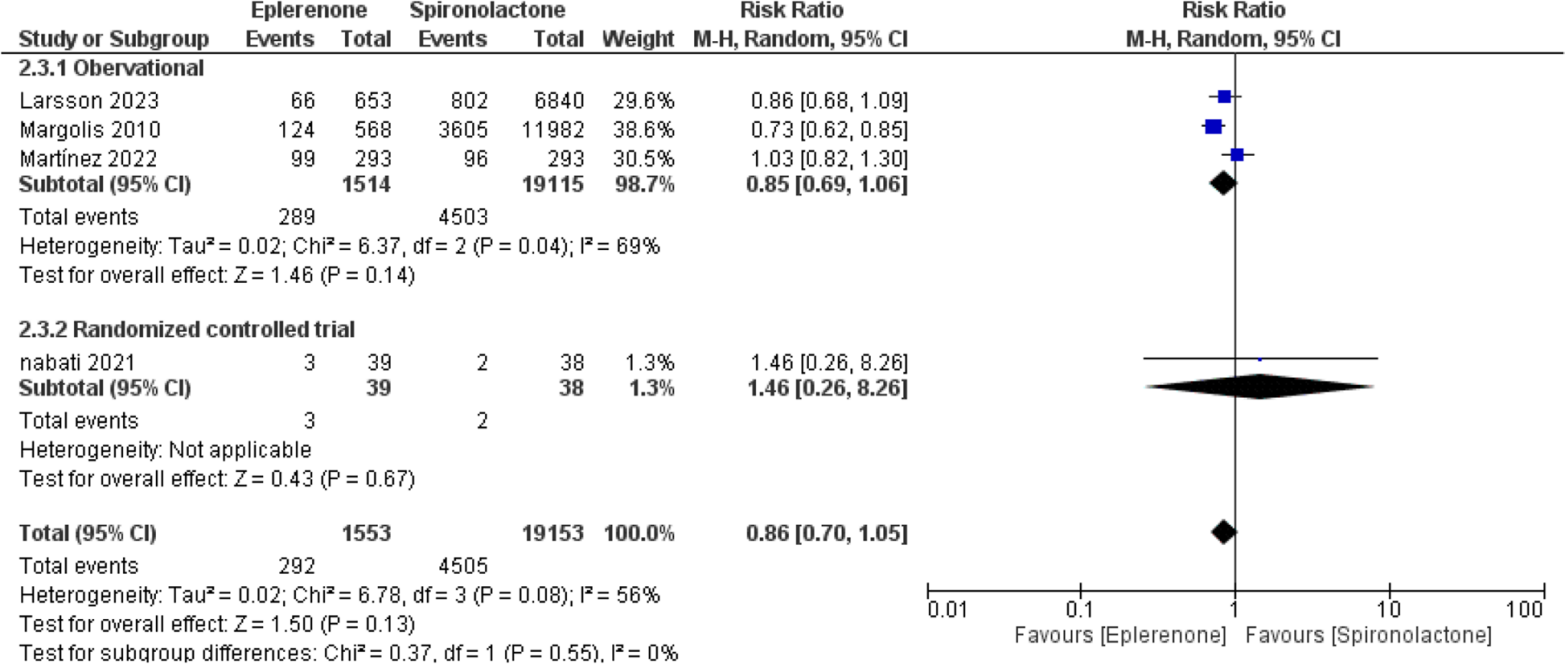
Forest plot of heart failure hospitalization, M-H: Mantel-Hanzel, CI: Confidence interval

Four studies accessed the HF hospitalization rates. The pooled RR demonstrated similar hospitalization rates within the two drugs (RR = 0.86; 95% CI [0.70, 1.05], *P* = 0.13,). Combined studies showed significant heterogeneity (I^2^ = 56%, *P* = 0.08), as outlined in Fig. 6*.*

The observed heterogeneity was best addressed by eliminating the Martinez et al. study [10]; the pooled analysis showed a non-significant heterogeneity (I^2^ = 0%, *P* = 0.38). The meta-analysis was done again and demonstrated that eplerenone was superior to spironolactone in reducing heart failure hospitalization rates compared to spironolactone (RR = 0.77; 95% CI [0.67, 88], *P* = 0.001), as outlined in Supplementary Fig. 5.

Additionally, the pooled RR remained statistically insignificant within the RCTs and observational subgroups (*P* = 0.55), as shown in Fig. 6.

### Tolerability outcomes

#### Treatment withdrawal

**Fig. 7:**
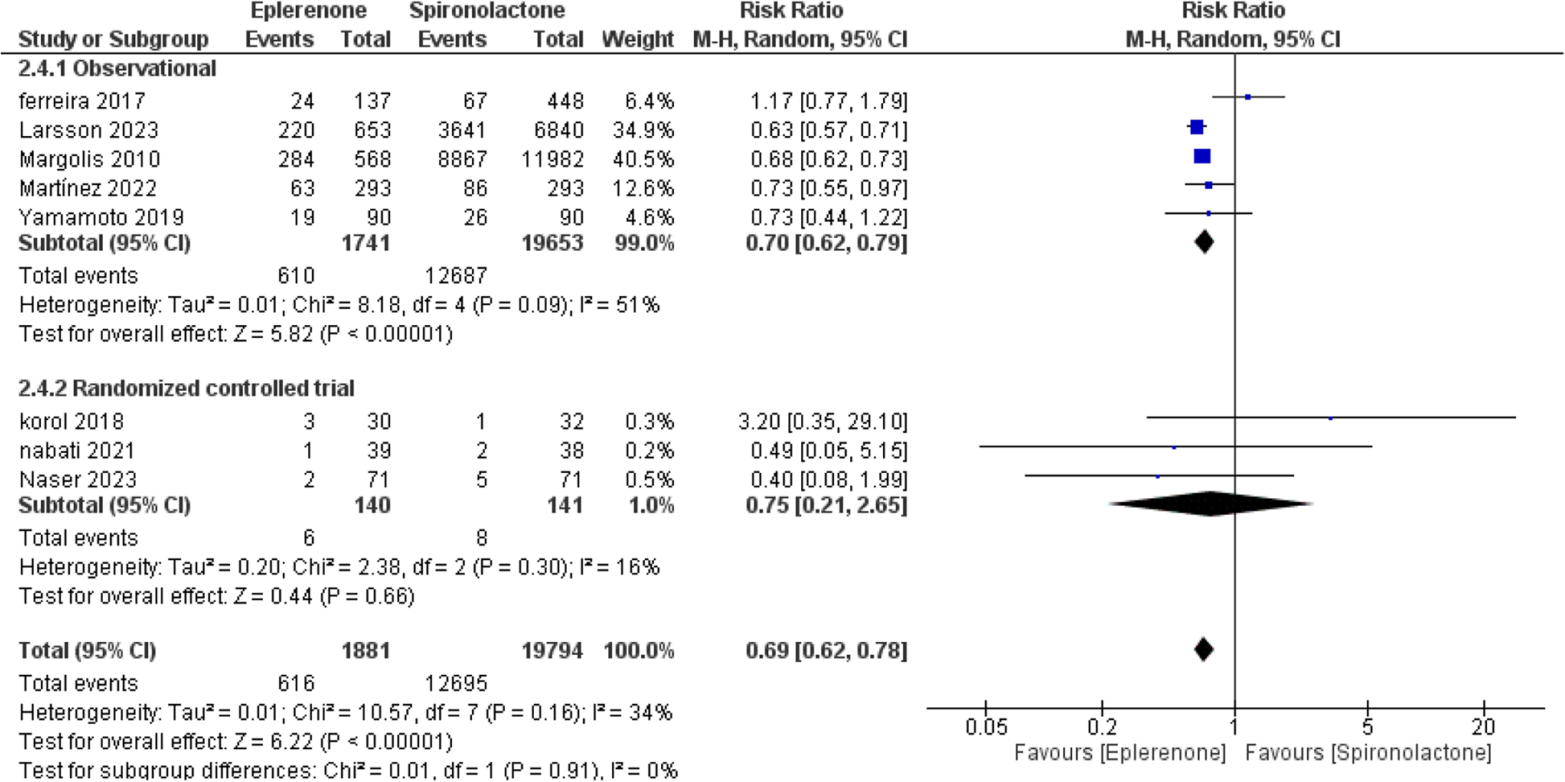
Forest plot of treatment withdrawal, M-H: Mantel-Hanzel, CI: Confidence interval

Eight studies accessed the treatment withdrawal. The overall RR demonstrated that eplerenone was linked to lower treatment withdrawal rates compared to spironolactone (RR = 0.69; 95% CI [0.62, 0.78], *P* = 0.001). Pooled results were associated with a non-significant heterogeneity (I^2^ = 34%, *P* = 0.16), as illustrated in Fig. 7*.*

In the observational studies subgroup, eplerenone was associated with lower treatment with-drawl rates than spironolactone (RR = 0.70; 95% CI [0.62, 0.79], *P* = 0.001). Inside the RCTs subgroup, the overall RR preferred neither (RR = 0.75; 95% CI [0.21, 2.65], *P* = 0.66), as outlined in Fig. 7.

### Treatment withdrawal due to side effects

The pooled RR showed that eplerenone significantly exhibited lower treatment withdrawal due to adverse effects rates compared to spironolactone (RR = 0.63; 95% CI [0.46, 0.85], *P* = 0.002). Pooled results did not reveal a significant heterogeneity (I^2^ = 0%, *P* = 0.74), as illustrated in Supplementary Fig. 6.

### Cross over

The pooled RR did not show any preference for either eplerenone or spironolactone (RR = 0.75; 95% CI [0.08, 7.12], P = 0.80). Pooled results were heterogenous (I^2^ = 99%, *P* = 0.001), as shown in Supplementary Fig. 7. Heterogeneity was effectively mitigated by excluding Margolis et al. study [22] (I^2^ = 18%, *P* = 0.30), and the meta-analysis was conducted again and demonstrated that eplerenone was associated with lower cross-over rates than spironolactone (RR = 0.35; 95% CI [0.23, 0.54], *P* = 0.001) as illustrated in Supplementary Fig. 8.

### Safety outcomes

#### Gynecomastia

**Fig. 8:**
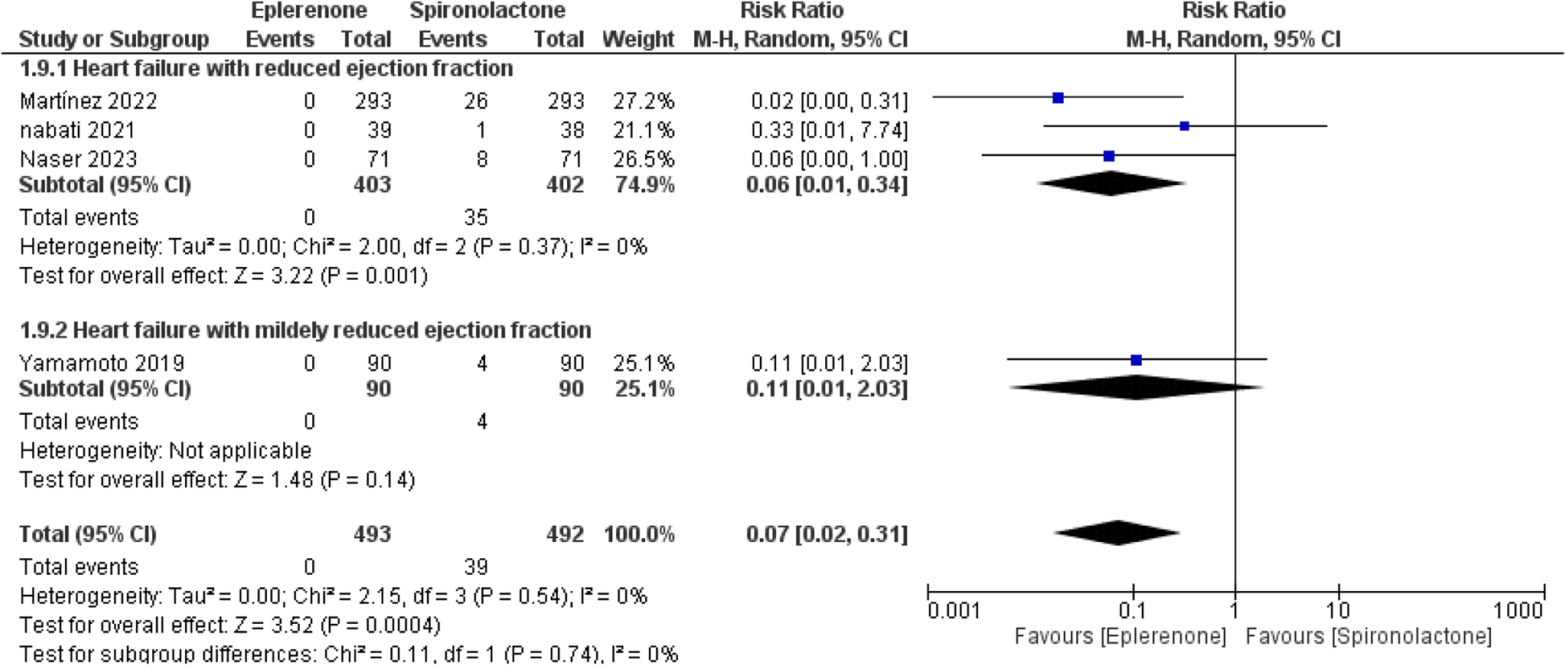
Forest plot of gynecomastia, M-H: Mantel-Hanzel, CI: Confidence interval

The pooled RR demonstrated that eplerenone decreased the risk of gynecomastia compared to spironolactone (RR = 0.07; 95% CI [0.02, 0.31], *P* = 0.004). Combined studies showed significant homogeneity (I^2^ = 0%, *P* = 0.54), as shown in Fig. 8.

Eplerenone showed lower gynecomastia rates than spironolactone in patients with HFrEF (RR = 0.06; 95% CI [0.01, 0.34], *P* = 0.001). Moreover, Within HFmrEF patients, the overall RR preferred neither (RR = 0.11; 95% CI [0.01, 2.03], *P* = 0.14), as illustrated in Fig. 8.

Regarding the observational studies, the overall effect favored eplerenone in decreasing the gynecomastia compared to eplerenone (RR = 0.04; 95% CI [0.01, 0.33], *P* = 0.002)*.* Eplerenone showed comparable gynecomastia rates compared to spironolactone within RCTs (RR = 0.13; 95% CI [0.02, 1.04], *P* = 0.05), as illustrated in Supplementary Fig. 9.

### Hyperkalemia

The overall RR did not show any preference for either of the two drugs (RR = 0.70; 95% CI [0.32, 1.53], *P* = 0.38). Pooled studies showed a non-significant heterogeneity (I^2^ = 57%, *P* = 0.07), as shown in Supplementary Fig. 10*.*

### Renal failure

The overall RR preferred neither (RR = 0.81; 95% CI [0.37, 1.78], *P* = 0.61). Pooled results were found to be homogenous (I^2^ = 0%, *P* = 0.51), as outlined in Supplementary Fig. 11*.*

### Hypotension

The overall RR did not prefer either of eplerenone or spironolactone (RR = 2.05; 95% CI [0.26, 16.21], *P* = 0.50). Pooled studies showed significant heterogeneity (I^2^ = 56%, *P* = 0.10), as illustrated in Supplementary Fig. 12*.*

## Discussion

### Key findings

Our meta-analysis investigated the efficacy and safety of eplerenone relative to spironolactone within the HF population. Our pooled analysis revealed that eplerenone outperformed spironolactone in mitigating the risk of all-cause mortality and cardiovascular mortality in HF patients. Interestingly, eplerenone showed lower all-cause mortality rates within HFrEF patients compared to spironolactone. However, Eplerenone and spironolactone showed comparable mortality rates in patients with HFmrEF. Additionally, eplerenone showed similar rates of HF hospitalization compared to spironolactone. Our study concluded that eplerenone exhibited lower treatment withdrawal rates than spironolactone, and there was no preference for either eplerenone or spironolactone regarding the cross-over rates. Furthermore, eplerenone was associated with a decreased risk of gynecomastia compared to spironolactone and no preference for eplerenone or spironolactone in terms of hyperkalemia, renal failure, and hypotension.

### Explanations of the findings

According to the current recommendations, the use of MRA, whether eplerenone or spironolactone, is advised to mitigate the incidence of HF hospitalization and mortality in symptomatic patients with HFrEF or HFmrEF [3]. Both medications showed constant reductions in both mortality and morbidity across diverse subsets of HFrEF patients [4, 7, 26]. Nevertheless, there is a lack of head-to-head comparisons between the two drugs in terms of efficacy, treatment tolerance, and safety endpoints. Our pooled analysis revealed lower all-cause and cardiovascular mortality with eplerenone relative to spironolactone, especially in patients with HFrEF. Martinez et al. [10] performed an observational single-center study with a total of 586 patients in Spain to evaluate the effectiveness and safety of eplerenone in comparison to spironolactone in patients with HFrEF. This study's findings aligned with our study as it concluded that eplerenone was linked to a lower incidence of all-cause and cardiovascular mortality compared to spironolactone. However, this study is limited by its observational, non-randomized nature, which may introduce some selection bias, and it was also performed in a specialized HF unit. Hence, its applicability and validity in other clinical settings remain uncertain. Naser et al. [11] performed an RCT with a total of 142 HFrEF patients in Bosnia and Herzegovina to compare eplerenone and spironolactone regarding the clinical and hemodynamic outcomes. They also concluded that eplerenone was superior to spironolactone in terms of all-cause and cardiovascular mortality in patients with HFrEF. Our study findings also aligned with this study.

To date, there is no proven explanation for the effectiveness of eplerenone in reducing mortality rates compared to spironolactone. An underlying explanation for this finding may be the different metabolic profiles between eplerenone and spironolactone, with spironolactone believed to have a poorer metabolic profile, potentially leading to divergent impacts on the cardiovascular risk profiles of patients treated with it [10]. Previous small RCT with a total of 107 HF patients concluded that spironolactone was correlated to a notable increase in cortisol and glycated hemoglobin and a substantial decrease in adiponectin; nevertheless, no notable change was observed in eplerenone-treated individuals [25]. Furthermore, there is emerging evidence indicating that the antiandrogenic properties of spironolactone might have negative consequences in HF, especially in men. Those patients usually suffered from anabolic deficiency, which is thought to be associated with worse outcomes. Experimental studies suggest that testosterone might offer a protective impact against cardiomyocyte apoptosis, a process impeded by spironolactone but unaffected by eplerenone [27, 28]. Furthermore, some emerging reports suggest that the actions of aldosterone are exhibited through slow genomic and rapid non-genomic mechanisms. Non-genomic effects encompass elevation in systematic vascular resistance, negative inotropic impacts in the myocardium, coronary vasoconstriction, and an improvement in angiotensin II-mediated vasoconstriction in coronary arteries. Interestingly, eplerenone demonstrates a greater degree of inhibition of the non-genomic effects of aldosterone relative to spironolactone [23].

Additionally, Larsson et al. [21] also conducted a large observational study with a total of 7493 on the Danish National Patient Registry in Denmark. This study assessed eplerenone compared to spironolactone in HFrEF patients, and it revealed comparable rates between the two drugs regarding all-cause mortality. This study is limited by its observational nature, which may cause some selection bias, and the patients in the eplerenone group were younger and more frequently treated with sacubitril/valsartan compared to patients in the spironolactone group. They also did not depend on ejection fraction when they identified their HFrEF patients; they used another validated approach to identify HFrEF patients. They classified the patients as HFrEF patients based on the prescription of renin-angiotensin-system inhibitors, and beta-blockers during the first 120 days subsequent HF diagnosis. Within HFmrEF patients, Eplerenone doesn’t show a significant superiority over spironolactone regarding all-cause mortality. Yamamoto et al. [12] conducted an observational study with a total of 180 propensity score-matched patients with HFmrEF. They evaluated eplerenone compared to spironolactone and suggested that there is no significant difference between the two drugs regarding all-cause mortality. However, this study is tempered by its small sample size and observational design.

Regarding HF hospitalization, our pooled analysis failed to show any significant difference between the two drugs regarding heart failure hospitalization, and the pooled studies were heterogeneous. Interestingly, after conducting a sensitivity analysis and excluding the Martinez et al. study [10], the heterogeneity was resolved, and the meta-analysis showed lower HF hospitalization rates with eplerenone compared to spironolactone. Martinez et al. showed comparable heart failure hospitalization rates between the two drugs. This finding can be explained by the observational nature of this study, and that repeated hospitalizations were not considered and analyzed in this study. Our results also showed that eplerenone was associated with lower treatment withdrawal rates compared to spironolactone. Larsson et al. [21] confirmed this crucial finding. Margolis et al. [22] performed a large retrospective observational study with a total of 12,550 HF patients. They evaluated the adherence and tolerability outcomes of eplerenone compared to spironolactone in HF patients. They concluded that eplerenone was associated with lower treatment-withdrawal rates when compared to spironolactone. However, this study is limited by its observational nature, and its population comprises employed or retried managed care beneficiaries, and it couldn’t be generalizable to the general population.

Eplerenone is thought to be associated with less dysmenorrhea in women and gynecomastia in men compared to spironolactone, which might constitute an obstacle for treatment discontinuation with eplerenone in real-world contexts [29, 30]. Moreover, our study didn’t show any significant difference between eplerenone and spironolactone regarding the cross-over and switching rates, and the pooled studies were heterogeneous. However, after removing Margolis et al. study [22], eplerenone showed lower crossover rates compared to spironolactone. Margolis et al. concluded that eplerenone was associated with higher crossover rates compared to spironolactone. The higher eplerenone cost could explain this finding. Our study also concluded lower gynecomastia rates with eplerenone in comparison to spironolactone. Martinez et al. [10] and Naser et al. [11] also agreed with this finding. Spironolactone (but not eplerenone) is thought to inhibit the free testosterone from binding to the androgen receptors, eventually leading to more estrogen binding to breast tissue proliferation, eventually resulting in gynecomastia [4, 26]. Our study also didn’t find any significant difference between the two drugs in other side effects like hyperkalemia, renal failure, and hypotension. However, our pooled analysis showed more favorable effects with eplerenone compared to spironolactone, eplerenone also has several disadvantages. Eplerenone showed 20-fold less in vitro affinity for mineralocorticoid receptors compared to spironolactone. Second, despite its advantageous safety profile, eplerenone is not widely used, primarily due to its cost and limited availability in many insurance formularies [31].

### Alignment with previous studies

None of the previous studies directly compared eplerenone and spironolactone, making this study the first systematic review and meta-analysis to directly compare these two drugs for the treatment of HF. However, some studies have indirectly compared these two drugs in patients with HF. Frankenstein et al. [32] conducted a network meta-analysis study with finally 14 included studies comparing the efficacy of different MRAs. Indirect comparisons between eplerenone and spironolactone showed comparable results in terms of all-cause mortality, cardiovascular mortality, and HF hospitalization. Interestingly, eplerenone showed lower gynecomastia rates when compared to other MRAs. Moreover, Yang et al. [33] also conducted a network meta-analysis, which finally included 13 studies. They concluded that finerenone is superior to Spironolactone and Eplerenone in decreasing the risk of cardiovascular mortality, hospitalization, and adverse events. A recent network meta-analysis executed by Pamporis et al. [34] evaluated the effect of MRAs in clinical endpoints in patients with HFrEF. With a finally included 32 RCTs, the network estimate between eplerenone and spironolactone did not reveal any notable difference regarding all-cause mortality (HR = 0.99, 95% CI (0.78, 1.24) and cardiovascular mortality (HR = 0.94, 95% CI (0.61, 1.44).

However, the pooled direct estimates showed that eplerenone was superior to spironolactone in reducing all-cause mortality (HR = 0.62, 95% CI 0.41, 0.94) and cardiovascular mortality (HR = 0.51, 95% CI (0.26, 0.98) in HFrEF patients, which agreed with our findings. Furthermore, eplerenone did not reveal any significant difference compared to spironolactone regarding heart failure hospitalization based on either the direct estimate or the network estimate. Nevertheless, regarding the treatment discontinuation and gynecomastia, the pooled network estimates showed a notable improvement with eplerenone compared to spironolactone, while the pooled direct estimates favored neither. Although The network design incorporates all available evidence, it provides less certainty than the direct comparison of interventions utilized in our study design. Additionally, it is worth noting that the direct estimate of their study was based on only two studies while we included more studies, and patents and evaluated more outcomes than any of the previous studies.

### Strengths and limitations of this study

To the best of our knowledge, our study is the first systematic review and meta-analysis to provide a pooled analysis for available direct comparisons between eplerenone and spironolactone in patients with HF. This meta-analysis included a notable number of studies and offered comprehensive results by utilizing evidence from RCTs and observational studies. Our study also included a notable number of patients, and the studies were carried out in different geographical regions, which supports our results' generalizability to the general population. However, this study faces some limitations. First, the observational nature of many studies that could introduce potential selection bias. Second, the inclusion of RCTs and observational studies in a combined analysis can potentially induce inconsistency, which may impact the strength of our results and the capacity to make definitive conclusions. To overcome these limitations, we conducted a subgroup analysis based on the study design. The RCTs and observational studies pooled effect estimates did not differ notably regarding the composite outcome of cardiovascular mortality or hospitalization and HF hospitalization. However, in terms of treatment withdrawal and gynecomastia, the RCTs pooled effect size did not exhibit any significant difference between the two drugs in contrast to the observational studies, as they showed a notable superiority for eplerenone over spironolactone concerning treatment withdrawal and gynecomastia. This finding could be attributed to the lower sample size and shorter follow-up durations of RCTs compared to the observational studies. Therefore, large RCTs with longer follow-up durations comparing the two drugs are needed. Third, the risk of bias of included RCTs demonstrated a moderate risk of bias, especially in the randomization phase, with only one study showing a high risk of bias. To overcome this limitation, we conducted a leave-one-out test to ensure the reliability of the pooled effect size. Fourth, our investigation encompassed RCTs with relatively small sample sizes, which could potentially restrict the statistical power to identify differences between eplerenone and spironolactone. Fifth, it is important to note that the mean duration of follow-up in the included studies is quite short (20 months), which may fail to adequately capture long-term outcomes and potential adverse effects. Finally, the notably lower all-cause mortality rates observed with eplerenone compared to spironolactone were primarily derived from a single study (Naser et al. study), which is an RCT with a relatively small sample size, so the final conclusions may be underpowered.

### Clinical implications and future suggestions

This meta-analysis suggests eplerenone as a preferable option for HF patients, particularly those with high mortality risk. Moreover, eplerenone should be preferred over spironolactone for patients with HFrEF, not HFmrEF patients, based on the results of our primary analysis. Owing to the lower gynecomastia rates observed with eplerenone compared to spironolactone, it is recommended to prescribe eplerenone to male patients. The current ACC / AHA guidelines recommend that MRAs (either eplerenone or spironolactone) should be prescribed for patients with HFrEF in order to mitigate the risk of mortality and morbidity [3]. However, they do not specifically recommend either eplerenone or spironolactone. Based on our results that favored eplerenone over spironolactone within the HFrEF population, particularly regarding mortality and safety outcomes, we recommended that eplerenone should be favored over spironolactone, especially for individuals with HFrEF. Furthermore, AHA / ACC guidelines recommend that MRAs (either eplerenone or spironolactone) could be considered for individuals with HFmrEF. However, within HFmrEF population, our meta-analysis did not find any notable differences between the two drugs, especially regarding mortality and safety endpoints. The future guidelines should address our findings carefully in order to provide the most effective and safe treatment for HF patients.

It underscores the need for further research to identify eplerenone's ideal indication criteria and to compare its long-term safety and efficacy with spironolactone. Future recommendations include conducting large, well-designed head-to-head trials between eplerenone and spironolactone in HF patients, with an emphasis on long-term outcomes, adverse event risks, and effectiveness in specific groups such as those with preserved ejection fraction, diabetes, and renal issues to refine treatment strategies.

## Conclusion

This meta-analysis provides evidence that eplerenone may be a more effective and better-tolerated treatment option for patients with heart failure than spironolactone. Eplerenone was associated with lower mortality, treatment withdrawal, and gynecomastia rates. However, eplerenone did not show any significant difference relative to spironolactone regarding HF hospitalization, renal failure, hyperkalemia, and hypotension. Further research, including head-to-head trials, is required to confirm our findings.

## Supplementary Information

Supplementary Material 1.

Supplementary Material 2.

## Data Availability

The datasets supporting the conclusions of this article are included within the article and its additional file.

## Abbreviations

HF: Heart failure
MRA: Mineralocorticoid receptor antagonist
ACC: American College of Cardiology
HFrEF: Heart failure with reduced ejection fraction
HFmrEF: Heart failure with mildly reduced ejection fraction
PRISMA: Preferred reporting items for systematic reviews and meta-analyses
ROB 2: Risk of bias tool two
NOS: Newcastle–Ottawa-scale
RCT: Randomized controlled trial
RR: Risk ratio
HR: Hazard ratio
CI: Confidence interval
I^2^: Inconsistency test

## Data availability

No datasets were generated or analysed during the current study.
