# Supplementary material for: Comparative effectiveness and safety of eplerenone and spironolactone in patients with heart failure: a systematic review and meta-analysis
Source: BMC Cardiovasc Disord. 2024 Sep 13;24:489. doi: 10.1186/s12872-024-04103-7 (PMC11395778; doi:10.1186/s12872-024-04103-7)
Supplement: Supplementary file 1 — Supplementary Material 1. [file 12872_2024_4103_MOESM1_ESM.docx]

| **Appendix A**: databases were used to search for articles related to the following key words: | | |
| --- | --- | --- |
| **Databases** | **Search Strategy** | **Results** |
| Pubmed | ("Eplerenone" OR "Eplerenon" OR "Inspra" OR "mineralocorticoid receptor antagonist" OR "Selara" OR "Epoxymexrenone" OR "aldosterone receptor antagonist") AND ("Spironolactone" OR "Spirolactone" OR "Veroshpiron" OR "Verospirone" OR "Spiractin" OR "Spirogamma" OR "Spirolang" OR "Aldactone" OR "Verospirone" OR "Aldactone A" OR "Aquareduct" OR "SC9420" ) AND ("Heart failure" OR "Cardiac Failure" OR "Heart failure with reduced ejection fraction" OR HFrEF OR "Systolic heart failure" OR "Heart failure with preserved ejection fraction" OR "HFpEF" OR "Diastolic heart failure") | 617 |
| Scopus | TITLE-ABS-KEY ( ( "eplerenone" OR "eplerenon" OR "inspra" OR "mineralocorticoidreceptorantagonist" OR "selara" OR "epoxymexrenone" OR "aldosterone receptor antagonist" ) AND ( "spironolactone" OR "spirolactone" OR "veroshpiron" OR "verospirone" OR "spiractin" OR "spirogamma" OR "spirolang" OR "aldactone" OR "verospirone" OR "aldactone a" OR "aquareduct" OR "sc9420" ) AND ( "heart failure" OR "cardiac failure" OR "heart failure with reduced ejection fraction" OR hfref OR "systolic heart failure" OR "heart failure with preserved ejection fraction" OR "hfpef" OR "diastolic heart failure" ) ) | 2542 |
| Web of science | ((ALL=(("Eplerenone" OR "Eplerenon" OR "Inspra" OR "mineralocorticoid receptor antagonist" OR "Selara" OR "Epoxymexrenone" OR "aldosterone receptor antagonist"))) AND ALL=(("Spironolactone" OR "Spirolactone" OR "Veroshpiron" OR "Verospirone" OR "Spiractin" OR "Spirogamma" OR "Spirolang" OR "Aldactone" OR "Verospirone" OR "Aldactone A" OR "Aquareduct" OR "SC9420" ))) AND ALL=(("Heart failure" OR "Cardiac Failure" OR "Heart failure with reduced ejection fraction" OR HFrEF OR "Systolic heart failure" OR "Heart failure with preserved ejection fraction" OR "HFpEF" OR "Diastolic heart failure")) | 846 |
| Cochrane library | ("Eplerenone" OR "Eplerenon" OR "Inspra" OR "mineralocorticoid receptor antagonist" OR "Selara" OR "Epoxymexrenone" OR "aldosterone receptor antagonist") AND ("Spironolactone" OR "Spirolactone" OR "Veroshpiron" OR "Verospirone" OR "Spiractin" OR "Spirogamma" OR "Spirolang" OR "Aldactone" OR "Verospirone" OR "Aldactone A" OR "Aquareduct" OR "SC9420" ) AND ("Heart failure" OR "Cardiac Failure" OR "Heart failure with reduced ejection fraction" OR HFrEF OR "Systolic heart failure" OR "Heart failure with preserved ejection fraction" OR "HFpEF" OR "Diastolic heart failure") in Title Abstract Keyword - (Word variations have been searched) | 366 |
| The total from the four databases: | | 4371 |
| Number of duplicates: | | 968 |
| Number after removing duplication:  (By Endnote): | | 3403 |

**Supplementary Table 1:** Search strategies and results for each database.
