# Supplementary material for: Comparative effectiveness and safety of eplerenone and spironolactone in patients with heart failure: a systematic review and meta-analysis
Source: BMC Cardiovasc Disord. 2024 Sep 13;24:489. doi: 10.1186/s12872-024-04103-7 (PMC11395778; doi:10.1186/s12872-024-04103-7)
Supplement: Supplementary file 2 — Supplementary Material 2. [file 12872_2024_4103_MOESM2_ESM.docx]

**Supplementary figures:**

**
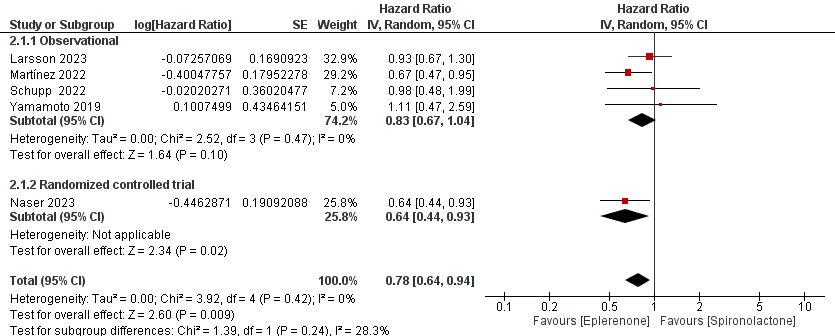
**

**Supplementary Figure 1:** Forest plot of all-cause mortality of the subgroup analysis according to study design, SE: Standard error, IV: Inverse-variance, CI: Confidence interval.


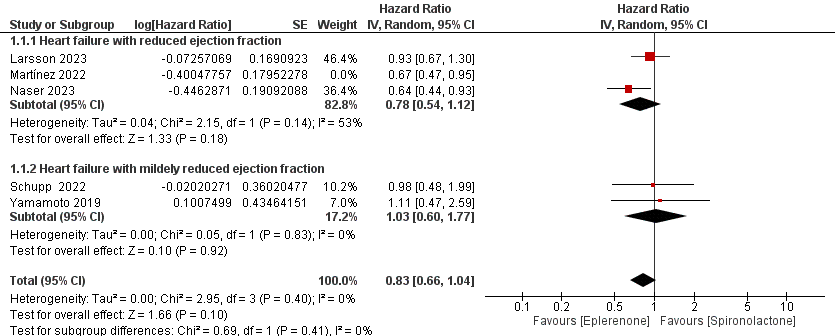


**Supplementary Figure 2:** Forest plot of all-cause mortality after conducting the Leave-one-out test, SE= standard error, IV: Inverse-variance, CI: Confidence interval.


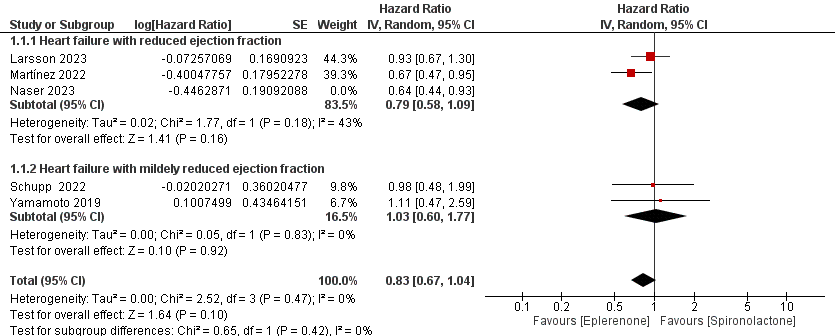


**Supplementary Figure 3:** Forest plot of all-cause mortality after conducting the Leave-one-out test, SE: Standard error, IV: Inverse-variance, CI: Confidence interval.


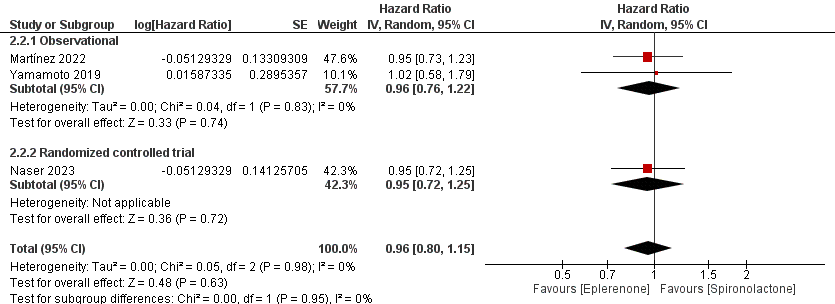


**Supplementary Figure 4:** Forest plot of the composite outcome of cardiovascular mortality or hospitalization based on subgroup analysis based on study design.


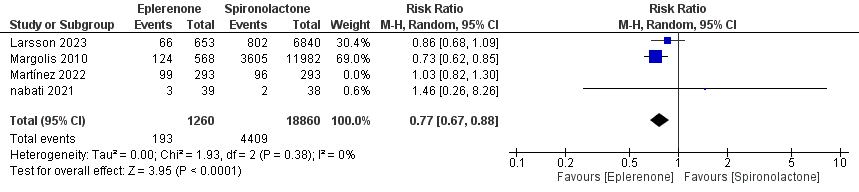


**Supplementary Figure 5:** Forest plot of heart failure hospitalization after conducting the sensitivity analysis, M-H: Mantel-Hanzel, CI: Confidence interval.


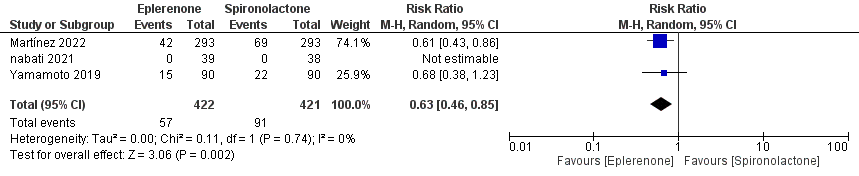


**Supplementary Figure 6:** Forest plot of treatment withdrawal due to adverse effects, M-H: Mantel-Hanzel, CI: Confidence interval.


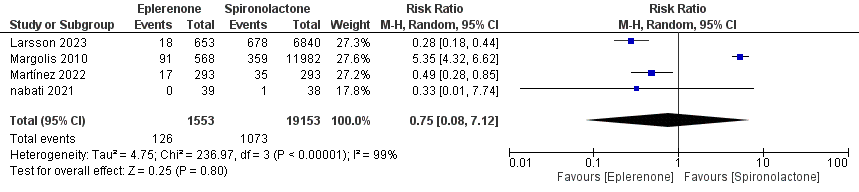


**Supplementary Figure 7:** Forest plot of cross-over, M-H: Mantel-Hanzel, CI: Confidence interval.


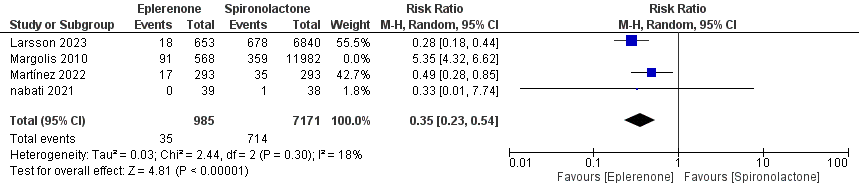


**Supplementary Figure 8:** Forest plot of cross-over after conducting the sensitivity analysis, M-H: Mantel-Hanzel, CI: Confidence interval.


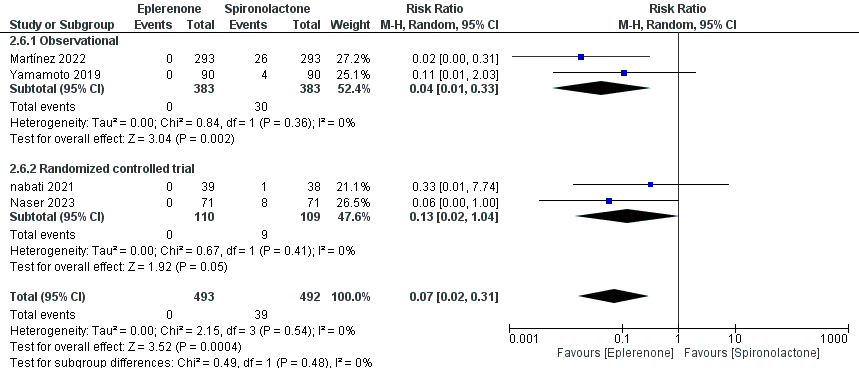


**Supplementary Figure 9:** Forest plot of gynecomastia after conducting a subgroup analysis based on study design, M-H: Mantel-Hanzel, CI: Confidence interval.


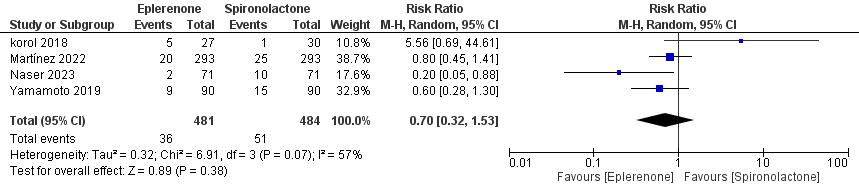


**Supplementary Figure 10:** Forest plot of hyperkalemia, M-H: Mantel-Hanzel, CI: Confidence interval.


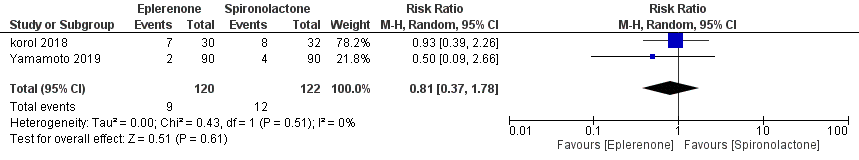


**Supplementary Figure 11:** Forest plot of renal failure, M-H: Mantel-Hanzel, CI: Confidence interval.


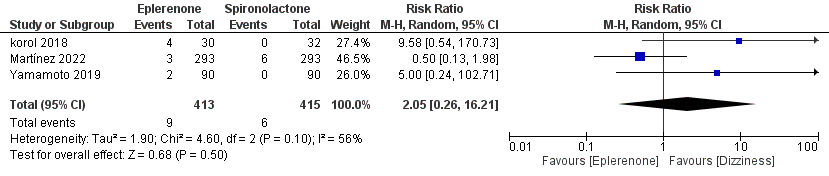


**Supplementary Figure 12:** Forest plot of hypotension, M-H: Mantel-Hanzel, CI: Confidence interval.
